# Red blood cell transfusion in the resuscitation of septic patients with hematological malignancies

**DOI:** 10.1186/s13613-017-0292-3

**Published:** 2017-06-12

**Authors:** Adrien Mirouse, Matthieu Resche-Rigon, Virginie Lemiale, Djamel Mokart, Achille Kouatchet, Julien Mayaux, François Vincent, Martine Nyunga, Fabrice Bruneel, Antoine Rabbat, Christine Lebert, Pierre Perez, Anne Renault, Anne-Pascale Meert, Dominique Benoit, Rebecca Hamidfar, Mercé Jourdain, Michaël Darmon, Elie Azoulay, Frédéric Pène, on behalf of the Groupe de Recherche sur la Réanimation Respiratoire en Onco-Hématologie (Grrr-OH)

**Affiliations:** 1 Réanimation médicale, hôpital Cochin, Assistance Publique-Hôpitaux de Paris (AP-HP) and Université Paris Descartes, 27 rue du Faubourg Saint-Jacques, 75014 Paris, France; 2 Département de biostatistiques, Hôpital Saint-Louis, AP-HP and Université Paris Diderot, Paris, France; 3 Réanimation médicale, Hôpital Saint-Louis, AP-HP and Université Paris Diderot, Paris, France; 4 Département d’anesthésie-réanimation, Institut Paoli-Calmettes, Marseille, France; 5 Réanimation médicale et médecine hyperbare, CHU d’Angers, Angers, France; 6 Réanimation médicale, Hôpital de la Pitié-Salpêtrière, AP-HP and Université Pierre et Marie Curie, Paris, France; 7 Réanimation polyvalente, Centre Hospitalier Intercommunal, Montfermeil, France; 8 Centre Hospitalier de Roubaix, Roubaix, France; 9 Réanimation polyvalente, Hôpital André Mignot, Le Chesnay, France; 10 Unité de soins intensifs respiratoires, Hôpital Cochin, AP-HP and Université Paris Descartes, Paris, France; 11 Réanimation polyvalente, Centre Hospitalier Départemental, La Roche-sur-Yon, France; 12 Réanimation médicale, Hôpital Brabois, Nancy, France; 13 Réanimation médicale, Centre Hospitalier de Brest, Brest, France; 14 Service des soins intensifs et urgences oncologiques, Institut Jules Bordet, Brussels, Belgium; 15 Ghent University Hospital, Ghent, Belgium; 16 Réanimation médicale, CHU Grenoble-Alpes, Grenoble, France; 17 Université de Lille and Réanimation Polyvalente, CHU de Lille, Lille, France; 18 Réanimation médicale, Centre Hospitalier de Saint-Etienne, Saint-Etienne, France

**Keywords:** Hematological malignancy, Severe sepsis, Septic shock, Anemia, Red blood cell transfusion

## Abstract

**Background:**

Indications for red blood cell (RBC) transfusion in septic acute circulatory failure remain unclear. We addressed the practices and the prognostic impact of RBC transfusion in the early resuscitation of severe sepsis and septic shock in patients with hematological malignancies.

**Methods:**

We performed a retrospective analysis of a prospectively collected database of patients with hematological malignancies who required intensive care unit (ICU) admission in 2010–2011. Patients with a main admission diagnosis of severe sepsis or septic shock were included in the present study. We assessed RBC transfusion during the first two days as part of initial resuscitation.

**Results:**

Among the 1011 patients of the primary cohort, 631 (62.4%) were admitted to the ICU for severe sepsis (55%) or septic shock (45%). Among them, 210 (33.3%) patients received a median of 2 [interquartile 1–3] packed red cells during the first 48 h. Hemoglobin levels were lower in transfused patients at days 1 and 2 and became similar to those of non-transfused patients at day 3. Early RBC transfusion was more likely in patients with myeloid neoplasms and neutropenia. Transfused patients displayed more severe presentations as assessed by higher admission SOFA scores and blood lactate levels and the further requirements for organ failure supports. RBC transfusion within the first two days was associated with higher day 7 (20.5 vs. 13.3%, p = 0.02), in-ICU (39 vs. 25.2%, p < 0.001) and in-hospital (51 vs. 36.6%, p < 0.001) mortality rates. RBC transfusion remained independently associated with increased in-hospital mortality in multivariate logistic regression (OR 1.52 [1.03–2.26], p = 0.03) and propensity score-adjusted (OR 1.64 [1.05–2.57], p = 0.03) analysis.

**Conclusions:**

RBC transfusion is commonly used in the early resuscitation of septic patients with hematological malignancies. Although it was preferentially provided to the most severe patients, we found it possibly associated with an increased risk of death.

**Electronic supplementary material:**

The online version of this article (doi:10.1186/s13613-017-0292-3) contains supplementary material, which is available to authorized users.

## Background

Septic shock is a frequent and dreaded complication in patients with malignancies. Following the overall improvement in the management of the disease, the last two decades have witnessed dramatic improvements in the prognosis of cancer patients presenting with severe sepsis and septic shock [1–4]. A major advance in the general management of sepsis was the implementation of aggressive resuscitation strategies, primarily inspired from the pivotal study by Rivers and colleagues, which promoted a protocol-guided hemodynamic resuscitation based on circulatory and tissue oxygenation objectives, the so-called early goal-directed therapy (EGDT) [5]. The genuine EGDT algorithm included a hematocrit target of 30% in case of persistent tissue hypoxia. Two-thirds of EGDT-treated patients therefore received red blood cell (RBC) transfusion within the early 72 h of resuscitation. However, the own prognostic value of RBC transfusion was not specifically assessed. Moreover, the study was performed in the late 1990s at the time when liberal fluid filling accounted for a high incidence of dilution anemia. All three recent replication studies of early goal-directed therapy retrieved lower requirements in RBC transfusion along with a more restrictive fluid filling policy [6–8]. Indications of RBC transfusion in the resuscitation of severe sepsis remain unclear [9], and it is noteworthy that the 2012 Surviving Sepsis Campaign guidelines were not able to provide any firm recommendation about the optimal transfusion threshold during hemodynamic instability [10]. Owing to the remaining controversy about optimal hemoglobin thresholds for transfusion in sepsis resuscitation, additional data are needed to optimize practices at the bedside.

Cancer patients with septic shock are at high risk of untractable multiple organ failure within the first days of ICU admission [11]. Early and aggressive resuscitation through restoration of both hemodynamics and tissue oxygenation is a major therapeutic goal in this setting. The optimal threshold for RBC transfusion and the eventual benefit in septic acute circulatory failure are debated. This issue appears particularly relevant to patients with hematological malignancies since the high prevalence of anemia imposed by malignant bone marrow infiltration or by cytotoxic treatments makes them particularly liable to urgent RBC transfusion in this setting in contrast to patients with delayed ICU-acquired anemia. This question was not specifically addressed by the leading trials about transfusion strategies in critically ill patients, since patients with chronic anemia were excluded from the pivotal TRICC trial and cancer patients were underrepresented in the TRISS trial [12–14]. We herein addressed the practices and the prognostic value of RBC transfusion as part of early sepsis resuscitation in a large cohort of patients with hematological malignancies who were admitted to the intensive care unit (ICU) for the main diagnosis of severe sepsis and septic shock.

## Patients and methods

### Study design

We conducted a substudy of a prospective, multicenter observational study that included 1011 consecutive adult patients with hematological malignancies who required ICU admission in 2010–2011. The study was approved by the appropriate ethics committees in France and Belgium. The methods and primary results of the study have been already published elsewhere [15]. The study involved 17 centers in France and Belgium, which may display different transfusion practices toward patients with hemodynamic instability. Blood banks followed similar national procedures for the collection, pre-storage leucoreduction, storage duration and delivery of RBC concentrates.

### Patients

We focused on patients with a primary diagnosis of severe sepsis or septic shock according to the common definitions of the Surviving Sepsis Campaign [10]. Briefly, severe sepsis was defined by a clinically or microbiologically documented infection associated with organ failure. Septic shock was defined as an acute circulatory failure requiring vasopressor support. We collected the requirements for RBC transfusion during the first 48 h as part of initial resuscitation, thereby identifying transfused and non-transfused patients.

The following data were prospectively collected: demographic features (age and gender), performance status prior to the acute complication, comorbidities using the Charlson comorbidity index, features of the underlying hematological malignancy (type of disease, time from diagnosis, status, hematopoietic stem cell transplantation). Newly diagnosed malignancies were defined as diagnosed within the past 4 weeks. The sepsis-related organ failure assessment (SOFA) score was computed on admission then daily throughout the stay in the ICU [16]. The features and management features of sepsis included the primary source of infection and the pathogen involved, as well as requirements for life-supporting interventions including vasopressor support, noninvasive/invasive mechanical ventilation and renal replacement therapy during the ICU stay.

### Statistical analysis

Quantitative variables were described as median [interquartile range] and categorical variables as number (percentage). The primary endpoint was vital status at hospital discharge. Multivariate logistic regression was used to assess the impact of day 1–2 transfusion on hospital mortality. The covariates identified as determinants of death in the primary analyses were entered into the model. Log-linearity was checked for continuous variables. Non-log-linear variables were dichotomised. Hosmer–Lemeshow goodness-of-fit tests were performed on multivariate regression models.

In order to limit bias in between-group comparisons, a propensity score-based approach was used to assess the impact of transfusion on hospital mortality, ICU mortality and 7-day mortality. The propensity score was defined as the probability that a patient with specific baseline characteristics receives transfusion. Then, two patients with identical propensity score values can be considered as comparable, and matching on the propensity score has been shown as one of the most efficient method for treatment effect assessment. We computed the propensity score using logistic regression to predict transfusion based on baseline characteristics known to be linked to the mortality and/or possibly to transfusion. Each subject treated by transfusion was randomly selected and then matched (without replacement) to the nearest untreated subject based on calipers of width of 0.2 of the standard deviation of the logit of the propensity score [17]. An inverse probability weighting approach for propensity score was also considered as sensitivity analysis. Missing data were handled using multiple imputation by chained equation. Fifty complete dataset were created using 10 iterations of the chained equation process. Rubin’s rules were applied after the evaluation of the treatment effect on each complete dataset.

All tests were two-sided and p values <0.05 were considered as indicating significant association. Analyses were performed using the R statistical software version 2.15.0 (http://www.Rproject.org).

## Results

**Fig. 1:**
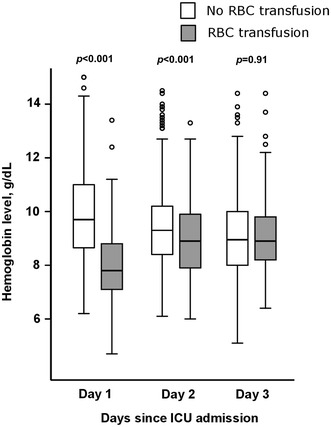
Hemoglobin levels in non-transfused (*n* = 421) and transfused (*n* = 210) patients. *ICU* intensive care unit, *RBC* red blood cell

Among the 1011 patients of the primary cohort, 347 (34.3%) were admitted to the ICU for severe sepsis and 284 (28%) for septic shock. Of those 631 patients, 210 (33%) received transfusions of 2 [1–3] packed red cells as part of the initial resuscitation strategy during the first two days in the ICU (148 (23.5%) and 89 (14.5%) at days 1 and 2, respectively). Among transfused patients, 6 (3%) presented concurrent bleeding at the time of admission. Hemoglobin levels were lower in transfused patients at day 1 (7.8 [7.1–8.8] vs. 9.7 [8.7–11] g/dL, p < 0.001) and day 2 (8.9 [7.9–9.9] vs. 9.3 [8.4–10.2] g/dL, p = 0.001) and became similar to those of non-transfused patients at day 3 (8.9 [8.2–9.8] vs. 8.9 [8–10]) (Fig. 1).

The other characteristics of non-transfused and transfused patients are shown and compared in Table 1. Early RBC transfusion was more likely in patients with acute myeloid leukemia and neutropenia. Transfused patients appeared with more severe presentations and tissue dysoxia as assessed by higher admission SOFA scores (8 [5–11] vs. 6 [4–9], p < 0.001), higher blood lactate levels (2.3 [1.5–4.9] vs. 2.0 [1.2–3.7] mmol/L, p = 0.02) (Additional file 1: Fig. S1) and the further requirements for organ failure supports including vasopressive drugs (70 vs. 57.7%, p = 0.004), invasive mechanical ventilation (55.2 vs. 48.7%, p = 0.01) and renal replacement therapy (30.5 vs. 22.2%, p = 0.03).

**Table 1:** Characteristics of patients without and with red blood cell transfusions at day 1/2

Characteristics	No RBC transfusion (*n* = 421)	RBC transfusion (*n* = 210)	*p*
Demographics			
Age (years)	60 [49–71]	60 [49–69]	0.68
Male gender	268 (63.7%)	138 (65.7%)	0.30
Comorbid illnesses			
Performance status 3–4	87 (20.7%)	51 (24.3%)	0.35
Charlson comorbidity index	4 [3–6]	4 [2–5]	0.77
Cardiovascular comorbidity	155 (36.8%)	77 (36.7%)	1
Coronary disease	31 (7.3%)	19 (9.0%)	0.53
Chronic heart failure	20 (4.8%)	14 (6.7%)	0.35
Peripheral arterial disease	19 (4.5%)	6 (2.8%)	0.39
Underlying malignancy			0.01
Non-Hodgkin lymphoma	149 (35.4%)	53 (25.2%)	
Hodgkin lymphoma	13 (3.1%)	2 (1.0%)	
Chronic lymphocytic leukemia	37 (8.8%)	13 (6.2%)	
Acute lymphocytic leukemia	23 (5.5%)	20 (9.5%)	
Acute myeloid leukemia	88 (20.9%)	69 (32.3%)	
Chronic myeloid leukemia	8 (1.9%)	4 (1.9%)	
Myeloma	56 (13.3%)	26 (12.4%)	
Myelodysplastic syndrome	23 (5.5%)	12 (5.7%)	
Others	24 (5.7%)	11 (5.2%)	
Time between diagnosis and ICU admission (days)	294 [38–1309]	199 [29–817]	0.06
Hematopoietic stem cell transplantation			
Autologous	50 (11.9%)	26 (12.4%)	0.72
Allogeneic	77 (18.3%)	33 (15.7%)	0.49
Malignancy status			
Newly diagnosed	120 (28.6%)	70 (33.3%)	0.95
Partial/complete remission	115 (27.5%)	54 (25.7%)	0.54
Time between hospital and ICU admissions (days)	6 [1–21]	8 [1–19]	0.38
ICU admission characteristics			
SOFA	6 [4–9]	8 [5–11]	<0.001
Hemoglobin (g/dL)	9.7 [8.7–11]	7.8 [7.1–8.8]	<0.001
Hemoglobin ranges [*n* (%)]			0.001
>9 g/dL	259 (66.1%)	44 (21.9%)	
7–9 g/dL	126 (32.1%)	117 (58.2%)	
<7 g/dL	7 (1.8%)	40 (19.9%)	
Platelet count (G/L)	77 [32–170]	31 [16–63]	<0.001
Neutropenia	122 (29.0%)	107 (51.0%)	<0.001
Lactate level (mmol/L)	2.0 [1.2–3.7]	2.3 [1.5–4.9]	0.02
Source of infection			
Pneumonia	252 (59.9%)	106 (50.5%)	0.03
Abdominal	15.7%	17.6%	0.61
Urinary tract	26 (6.2%)	10 (4.8%)	0.59
Catheter-related	5.0%	3.3%	0.46
Pathogens			
Gram-negative bacteria	108 (25.7%)	67 (31.9%)	0.12
Gram-positive bacteria	60 (14.3%)	21 (10.0%)	0.69
*Aspergillus*	46 (10.9%)	23 (11.0%)	1.00
*Pneumocystis*	20 (4.8%)	2 (1.0%)	0.02
Life-supporting interventions			
Vasopressive drugs	243 (57.7%)	147 (70%)	0.004
Invasive ventilation	205 (48.7%)	116 (55.2%)	0.01
Renal replacement therapy	92 (22.2%)	62 (30.5%)	0.03
Platelet transfusions			
Day 1	70 (16.6%)	96 (45.7%)	<0.001
Day 2	58 (13.7%)	82 (39.0%)	<0.001
Mortality			
ICU mortality	106 (25.2%)	82 (39.0%)	<0.001
Hospital mortality	154 (36.6%)	107 (51.0%)	<0.001

*ICU* intensive care unit, *RBC* red blood cell, *SOFA* sequential organ failure assessment

**Fig. 2:**
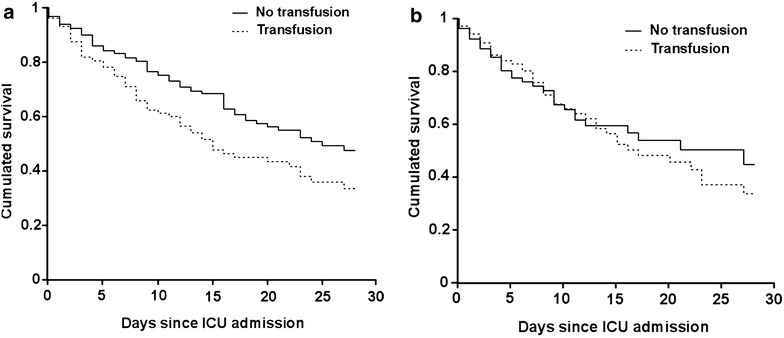
Survival estimates in non-transfused and transfused patients. Crude (**a**) and propensity score-adjusted (**b**) survival estimates in patients who did or did not receive red blood cell transfusion during the first two days of resuscitation. **a** Encompasses the whole cohort (631 patients distributed into 421 non-transfused and 210 transfused). **b** Includes 142 transfused patients with 142 propensity score-matched non-transfused counterparts. *ICU* intensive care unit

The overall in-ICU and in-hospital mortality rates were 28.9 and 41.4%, respectively. In univariate analysis, RBC transfusion within the first 48 h was associated with increased mortality at day 7 (20.5 vs. 13.3%, p = 0.02), in the ICU (39 vs. 25.2%, p < 0.001) and in the hospital (51 vs. 36.6%, p < 0.001) (Table 2; Fig. 2a). After adjustment with other relevant variables including the hemoglobin level on ICU admission and septic shock, RBC transfusion was no longer associated with 7-day mortality (OR 1.11 [0.66–1.87], p = 0.7), but tended to be associated with higher in-ICU and in-hospital mortality rate (OR 1.44 [0.94–2.21], p = 0.09 and OR 1.52 [1.03–2.26], p = 0.03, respectively). Other variables associated with in-hospital mortality were poor performance status, severity on admission as assessed by SOFA score, allogeneic hematopoietic stem cell transplantation, increased time to ICU admission and invasive aspergillosis. Partial or complete remission was associated with improved survival (Table 3).

**Table 2:** Characteristics of in-hospital survivors and deceased

Characteristics	In-hospital survivors (*n* = 370)	In-hospital deceased (*n* = 261)	*p*
Demographics			
Age (years)	58.5 [47–67.75]	63 [52–71]	0.01
Male gender	240 (64.9%)	166 (63.6%)	0.81
Comorbid illnesses			
Performance status 3–4	61 (16.5%)	77 (29.5%)	0.0001
Charlson comorbidity index	4 [2–5]	4 [3–6]	0.008
Underlying malignancy			0.90
Lymphoid disease	179 (48.4%)	131 (50.2%)	
Myeloid disease	121 (32.7%)	83 (31.8%)	
Time between diagnosis and ICU admission (days)	91 [14; 444]	90 [11; 396]	0.58
Hematopoietic stem cell transplantation			0.04
Autologous	52 (14.1%)	24 (9.2%)	
Allogeneic	55 (14.9%)	55 (21.1%)	
Malignancy status			0.019
Newly diagnosed	106 (28.7%)	84 (32.2%)	
Partial/complete remission	116 (31.4%)	53 (21.4%)	
Time between hospital and ICU admissions (days)	4 [0–16]	9 [1–25]	<0.0001
ICU admission characteristics			
SOFA score	6 [4–8]	8 [5–11.75]	<0.0001
Neutropenia	125 (33.8%)	104 (39.9%)	0.14
Lactate (mmol/L)	1.9 (1.125–3.575)	2.4 [1.6–5.6]	0.0001
Hemoglobin level (g/dL)			
Day 1	9.2 [8–10.7]	8.9 [7.8–10.2]	0.02
Day 2	9.2 [8.3–10.2]	9.1 [8.3–10]	0.33
Day 3	9 [8.1–10]	8.9 [8.2–9.975]	0.88
Day 7	8.8 [8.1–9.8]	8.8 [8.3–9.95]	0.53
RBC transfusion			
Day 1 and/or 2	103 (27.8%)	107 (41%)	0.0008
Day 3	42 (12.4%)	39 (17.3%)	0.13
Day 7	16 (10.6%)	21 (16.7%)	0.19
Source of infection			
Pneumonia	190 (51.4%)	68 (64.3%)	0.002
Abdominal	69 (18.7%)	34 (13.0%)	0.076
Urinary tract	21 (5.7%)	15 (5.8%)	1.00
Catheter-related	21 (5.7%)	7 (2.7%)	0.11
Pathogens			
Gram-negative bacteria	101 (27.3%)	74 (28.4%)	0.84
Gram-positive bacteria	56 (15.1%)	25 (9.6%)	0.053
*Aspergillus*	27 (7.3%)	42 (16.1%)	0.0008
*Pneumocystis*	17 (4.6%)	5 (1.9%)	0.11
Life-supporting interventions			
Vasopressive drugs	184 (49.7%)	206 (78.9%)	<0.0001
Invasive ventilation	124 (33.5%)	197 (75.5)	<0.0001
Renal replacement therapy	56 (15.4%)	98 (38.7%)	<0.0001

*ICU* intensive care unit, *RBC* red blood cell, *SOFA* sequential organ failure assessment

**Table 3:** Determinants of hospital mortality: multivariate analysis

Characteristics	Odds ratio (95% CI)	*p*
RBC transfusion at day 1/2	1.52 (1.03–2.26)	0.03
Hemoglobin level at ICU admission	1.0 (0.95–1.06)	0.87
Performance status 3–4	1.97 (1.29–3.02)	0.002
Lactate level at ICU admission	1.01 (0.96–1.05)	0.79
SOFA score at ICU admission	1.14 (1.1–1.21)	<0.001
Septic shock	1.30 (0.86–1.96)	0.21
Allogeneic HSCT recipient	2.14 (1.28–3.57)	0.004
Remission	0.53 (0.34–0.84)	0.007
Time between hospital and ICU admissions >1 day	1.69 (1.14–2.51)	0.009
Invasive aspergillosis	1.91 (1.08–3.4)	0.02

All variables entered into the model appear in the table. Hosmer–Lemeshow goodness of fit of the multivariate model was tested on each imputed dataset, with p values ranging from 0.22 to 0.96

*CI* confidence interval, *HSCT* hematopoietic stem cell transplantation, *ICU* intensive care unit, *RBC* red blood cell, *OR* odds ratio, *SOFA* sequential organ failure assessment

In order to refine the statistical adjustment between non-transfused and transfused patients, we built a propensity score of being transfused during the first two days following ICU admission. The propensity score was based on hemoglobin level at the time of ICU admission and on the following characteristics: age, gender, performance status, days since hospital admission, allogeneic hematopoietic stem cell transplantation, hematological malignancy type and status, time between its diagnosis and ICU admission, time between hospital and ICU admissions, neutropenia, aspergillosis, chemotherapy, admission SOFA score and septic shock. This score allowed matching 142 patients who did receive RBC transfusion as part of the initial resuscitation to 142 counterparts who did not. As shown in Fig. 2b, propensity score-matched transfused and non-transfused patients displayed similar survival trends (OR for in-hospital death 1.25 [0.75–2.05], p = 0.39). When including all 631 patients in a weighting analysis, day 1/2 RBC transfusion was still associated with increased hospital mortality (OR 1.64 [1.05–2.57], p = 0.03).

## Discussion

There is no doubt that the prognosis of critically ill patients with hematological malignancies has dramatically improved, and admission to the ICU is now viewed as a bridge to cure rather than a terminal process. The reasons for this trend are multiple, including general improvements in the prognosis of cancer, better and early identification of patients likely to benefit from intensive care and advances in the management of acute life-threatening disorders [18]. However, severe infections resulting in shock and/or acute respiratory failure represent dreaded complications in cancer patients and still account for a large number of deaths regardless of the stage of malignancy. It is clear that cancer patients already benefited from advances in the field of sepsis, but it remains a major area for improvement for this vulnerable population. Nevertheless, such immunocompromised patients were either underrepresented or even excluded from the leading clinical trials in severe sepsis and septic shock. Whether they are entitled to strictly similar management as non-immunocompromised patients is questionable.

The poor prognosis of hematological patients with severe sepsis is commonly attributed to the underlying immune defects resulting in impaired and delayed pathogen clearance, but additional mechanisms related to the disease itself or to its treatment may also worsen organ failures. For instance, endothelial or cardiac toxicity by chemotherapy and radiotherapy may contribute to the pathophysiology of acute circulatory failure [19], while lactic acidosis may not only reflect systemic tissue hypoperfusion but might also result from altered metabolism of tumor cells. However, intensity and duration or vasopressor support appear quite similar in cancer patients, either untreated or treated with chemotherapy, and in patients free of cancer but harboring alternative comorbidities [20]. Furthermore, microvascular derangements have been reported in non-septic patients with chemotherapy-induced neutropenia, albeit microcirculatory alterations induced by septic shock appeared quite similar in neutropenic and non-neutropenic patients [21]. Most importantly, cancer patients commonly display chronic anemia likely to contribute to the impaired oxygen delivery encountered in severe sepsis and septic shock and appear then liable to RBC transfusion early in the course of hemodynamic resuscitation.

The general practice of RBC transfusion in the ICU is guided by the apparently opposite objectives of preserving an appropriate tissue oxygenation while minimizing the number of packed red cells transfused. Studies investigating RBC transfusion in the ICU have so far been performed in general populations in which anemia is mostly acquired during the ICU stay as a result of dilution and blood loss, associated with an impaired erythropoietic response. In 1999, the pivotal TRICC study by Hebert and colleagues showed that a restrictive strategy of non-leucodepleted RBC transfusion to maintain hemoglobin above 7 g/dL was at least as effective as a liberal transfusion strategy aimed to maintain hemoglobin >10 g/dL in critically ill patients [12]. As of today, the recommendations for RBC transfusion in the ICU remain largely based on this study. Of note, this study excluded patients with a previous history of chronic anemia.

However, some studies challenged the general implementation of this restrictive strategy and suggested that a higher transfusion threshold might be beneficial in septic patients for whom oxygen delivery is of paramount importance. This was first derived from the study by Rivers and colleagues, in which most EGDT-treated patients had received RBC transfusion to maintain a hematocrit level above 30% [5]. Since then, three studies using propensity-adjusted analysis also reported that RBC transfusion was associated with improved survival in septic shock [22–24]. Furthermore, a Brazilian monocenter randomized study reported that a liberal RBC transfusion strategy (hemoglobin >9 g/dL) versus a restrictive strategy (hemoglobin >7 g/dL) improved survival in critically ill patients admitted to the ICU following major cancer surgery [25]. The reasons for this finding remain intriguing, but possibly related to a lower incidence of both cardiovascular complications and superinfections. The same team reported a benefit from a liberal transfusion strategy applied throughout the ICU stay in solid cancer patients with septic shock [26]. A multicenter randomized Scandinavian study addressed the transfusion policy in septic shock patients, of whom 7.5% had hematological malignancies and 9.5% had metastatic cancer. Patients were randomized to either a restrictive or a liberal transfusion policy to maintain hemoglobin levels higher than 7 or 9 g/dL, respectively [13]. Similar survival rates were reported in the restrictive and liberal randomization arms. Subgroup analysis in patients with hematological malignancies or metastatic cancer also retrieved similar survival rates in both randomization arms [27]. However, it should be emphasized that most patients had already been efficiently resuscitated and restored tissue oxygenation at the time of randomization. Although this study suggests that a restrictive transfusion policy can be safely implemented in most patients with septic shock, we do think that it does not provide a definite answer for patients with persistent and/or marked circulatory failure.

The trend in increased mortality in transfused patients is the most intriguing result of our study. Although this is in line with several case–control studies which suggested that RBC transfusion was associated with higher mortality and increased incidence of ICU-acquired complications in critically ill patients, the interpretation of this finding deserves caution. On one hand, this could suggest that requirements of RBC transfusion, presumably imposed by persistent tissue dysoxia, could represent a very potent prognostic factor even when adjusted to the classical determinants of death in this setting. On the other hand, there are considerable interindividual variations in the microcirculatory and tissue oxygenation responses to RBC transfusion [28, 29]. Increase in blood viscosity by RBC transfusion and storage lesions can result in paradoxical impairment on microcirculation and tissue oxygenation. While not being immediately fully efficient for oxygen delivery to tissue, red cells may then sludge within capillaries, interact with endothelial cells and promote inflammatory processes [30, 31]. However, recent studies using leucodepleted packed red cells did not retrieve any impact of storage duration on restoration of tissue oxygenation or survival status in critically ill patients with anemia [32, 33]. Finally, RBC transfusion may represent a risk factor for hospital-acquired infection as a result of transfusion-induced immunomodulation [34, 35]. Although we could not reliably collect the incidence of ICU-acquired complications, it is noteworthy that the non-adjusted survival curves of transfused and non-transfused patients forked as early as three days after ICU admission, suggesting that the poor impact of RBC transfusion was related to early events rather than secondary ICU-acquired complications.

One strength of this study is the prospective collection of data, although it was a secondary analysis of a database that was not designed to this specific aim. In general, data from the first three days were complete and accurate, but data collected later on were less detailed. Thus, the estimation of packed red cells transfused within the first three days was accurate, whereas daily transfusions thereafter were not collected. Nonetheless, neither the volume of fluid loading nor the fluid balance was accurately recorded in the database. In the same way, the incidence of delayed ICU-acquired infectious and non-infectious complications and the definite causes of death could not be reliably estimated. In the absence of guidelines, indications of RBC transfusion in this setting were probably quite inconsistent across centers. Hemoglobin levels in the RBC transfusion group were often higher than the recommended 7 g/dl threshold. However, this was an observational study reflecting routine practice. With respect to the lower hemoglobin levels and the severity of circulatory dysfunctions in transfused patients, it is likely that physicians followed a common pragmatic decision-making process at the bedside, based not only on a sole hemoglobin level but also on markers of tissue dysoxia. However, such an observational study can only provide exploratory data and statistical link, but is no substitute to prospective interventional studies.

## Conclusion

RBC transfusions are frequently used as part of the initial resuscitation of severe sepsis or septic shock in patients with hematological malignancies. Although RBC transfusion was preferentially administrated to the most severe patients, we found it possibly associated with an increased risk of death. Since the definite indications of RBC transfusions in resuscitation of severe sepsis remain questionable, cancer patients with a high prevalence of underlying anemia represent a relevant subgroup to address this question in a prospective manner.

## Additional file

**Additional file 1: Fig. S1.** Arterial lactate levels in non-transfused and transfused patients. RBC (red blood cell).

## Abbreviations

EGDT: early goal-directed therapy
HSCT: hematopoietic stem cell transplantation
ICU: intensive care unit
RBC: red blood cell
SOFA: sepsis-related organ failure assessment
