# Supplementary figures and images for: Red blood cell transfusion in the resuscitation of septic patients with hematological malignancies
Source: Ann Intensive Care. 2017 Jun 12;7:62. doi: 10.1186/s13613-017-0292-3 (PMC5468360; doi:10.1186/s13613-017-0292-3)

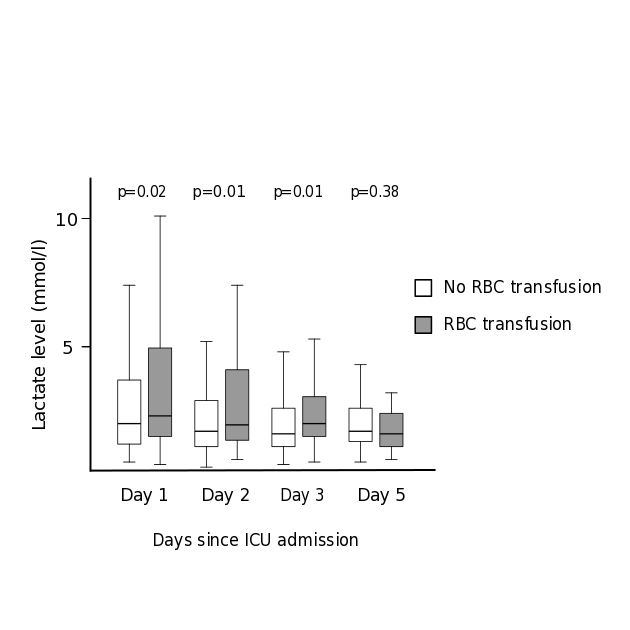

Supplement: Supplementary file 1 — Additional file 1: Fig. S1. Arterial lactate levels in non-transfused and transfused patients. RBC (red blood cell). [file 13613_2017_292_MOESM1_ESM.tif]
